# Genetic evidence supporting the causal role of gut microbiota in chronic kidney disease and chronic systemic inflammation in CKD: a bilateral two-sample Mendelian randomization study

**DOI:** 10.3389/fimmu.2023.1287698

**Published:** 2023-11-02

**Authors:** Feihong Ren, Qiubai Jin, Qi Jin, Yiyun Qian, Xuelei Ren, Tongtong Liu, Yongli Zhan

**Affiliations:** ^1^ Guang’anmen Hospital, China Academy of Chinese Medical Sciences, Beijing, China; ^2^ Graduate School, Beijing University of Chinese Medicine, Beijing, China; ^3^ Peking Union Medical College Hospital, Chinese Academy of Medical Science, Beijing, China

**Keywords:** two-sample Mendelian randomization, gut microbiota, chronic kidney disease, inflammation, causality two-sample mendelian randomization, causality

## Abstract

**Background:**

The association of gut microbiota (GM) and chronic kidney disease (CKD), and the relevancy of GM and chronic systemic inflammation in CKD, were revealed on the basis of researches on gut–kidney axis in previous studies. However, their causal relationships are still unclear.

**Objective:**

To uncover the causal relationships between GM and CKD, as well as all known GM from eligible statistics and chronic systemic inflammation in CKD, we performed two-sample Mendelian randomization (MR) analysis.

**Materials and methods:**

We acquired the latest and most comprehensive summary statistics of genome-wide association study (GWAS) from the published materials of GWAS involving GM, CKD, estimated glomerular filtration rate (eGFR), c-reactive protein (CRP) and urine albumin creatine ratio (UACR). Subsequently, two-sample MR analysis using the inverse-variance weighted (IVW) method was used to determine the causality of exposure and outcome. Based on it, additional analysis and sensitivity analysis verified the significant results, and the possibility of reverse causality was also assessed by reverse MR analysis during this study.

**Results:**

At the locus-wide significance threshold, IVW method and additional analysis suggested that the protective factors for CKD included family *Lachnospiraceae* (*P*=0.049), genus *Eubacterium eligens* group (*P*=0.002), genus *Intestinimonas* (*P*=0.009), genus *Streptococcu* (*P*=0.003) and order *Desulfovibrionales* (*P*=0.001). Simultaneously, results showed that genus *LachnospiraceaeUCG010* (*P*=0.029) was a risk factor for CKD. Higher abundance of genus *Desulfovibrio* (*P*=0.048) was correlated with higher eGFR; higher abundance of genus *Parasutterella* (*P*=0.018) was correlated with higher UACR; higher abundance of class *Negativicutes* (*P*=0.003), genus *Eisenbergiella* (*P*=0.021), order *Selenomonadales* (*P*=0.003) were correlated with higher CRP levels; higher abundance of class *Mollicutes* (0.024), family *Prevotellaceae* (*P*=0.030), phylum *Tenericutes* (*P*=0.024) were correlated with lower levels of CRP. No significant pleiotropy or heterogeneity was found in the results of sensitivity analysis, and no significant causality was found in reverse MR analysis.

**Conclusion:**

This study highlighted associations within gut-kidney axis, and the causal relationships between GM and CKD, as well as GM and chronic systemic inflammation in CKD were also revealed. Meanwhile, we expanded specific causal gut microbiota through comprehensive searches. With further studies for causal gut microbiota, they may have the potential to be new biomarkers for targeted prevention of CKD and chronic systemic inflammation in CKD.

## 1 Introduction

Recently, a worldwide trend of increasing prevalence has appeared in chronic kidney disease (CKD) (1). The most important and commonly used CKD-classifying quantitative traits include estimated glomerular filtration rate (eGFR) and urinary albumin-to-creatinine ratio (UACR) (2, 3). Based on these traits, an epidemiological investigation revealed (4) that the prevalence of CKD is estimated to be as high as 13.4% (11.7-15.1%) worldwide. What’s more, CKD significantly increases the mortality of patients, and heavily burdens the financial and healthcare system (5). In addition, Patients with CKD are usually in a chronic inflammatory status, as manifested by the elevated levels of inflammatory markers in CKD patients, which are caused by multiple factors (6). To some extent, renal functions and severity of inflammation in CKD patients can be reflected by levels of c-reactive protein (CRP) or other inflammatory markers (7). Interestingly, levels of the same inflammatory markers are inconsistent within the same period of CKD patients. Besides explicit risk factors including diabetes mellitus (DM) (8), hypertension (HTN) (9), and oxidative st (10) that can influence the inflammation of CKD patients, more risk factors remain to be explored.

Targeting explicit risk factors, the only interventions available for improving renal function and chronic systemic inflammation in patients with CKD are lifestyle changes, pharmacotherapy, and optimization of dialysis conditions (11). However, these interventions are either costly or lack sufficient clinical evidence to support their therapeutic effects. Hence, it is essential to identify potential risk factors for CKD and chronic systemic inflammation in CKD, and develop new preventive and therapeutic measures for them.

Gut microbiota (GM) is a crucial regulator of human health (12). As a critical regulator of human health, it has gradually received attention recently for being considered one of the new potential risk factors of CKD and chronic systemic inflammation in CKD (13, 14). Meijers et al. (15), in 2011, proposed the concept known as gut-kidney axis, revealing that GM might interact with CKD and chronic systemic inflammation in CKD through it. Lau Wei Ling et al. (16) further indicated that GM of CKD patients appeared to be ecologically imbalanced, and the degree of GM imbalance directly influenced the severity of inflammation in the dialysis population. Some studies asserted that interventions on GM could reduce the levels of inflammatory factors and delay disease progression in CKD patients (17). Conversely, some studies suggested that altering types and abundance of GM could not improve renal function and chronic systemic inflammation in CKD patients (18). Nevertheless, their findings were limited by confounding bias, small sample size and reverse causality. Therefore, the association effects they showed were not equivalent to causality. The causal relationships between GM and CKD, as well as GM and chronic systemic inflammation in CKD are still unclear.

Mendelian randomization (MR), as a novel genetic statistical method, can be an effective alternative to traditional epidemiological study approaches. It uses genetic variants strongly associated with exposure factors as instrumental variables (IVs) to statistically evaluate the causality of exposure and outcome (19). The advantage of MR over traditional epidemiological study approaches is that its random assignment method is determined by the DNA genotype. Thus, the influence of external factors on the robustness of causality can be limited to the greatest extent possible (20). In this research, the latest genome-wide association study (GWAS) statistics were used in two-sample MR analysis, and four sets of causal relationships were analyzed at the genetic level: GM and CKD, GM and eGFR, GM and UACR, GM and CRP. Also, we determined the specific causal GM in these four sets. The results supplemented gaps in existing research and might provide new ideas for improving and enriching treatment measures.

## 2 Materials and methods

### 2.1 Study design

**Figure 1:**
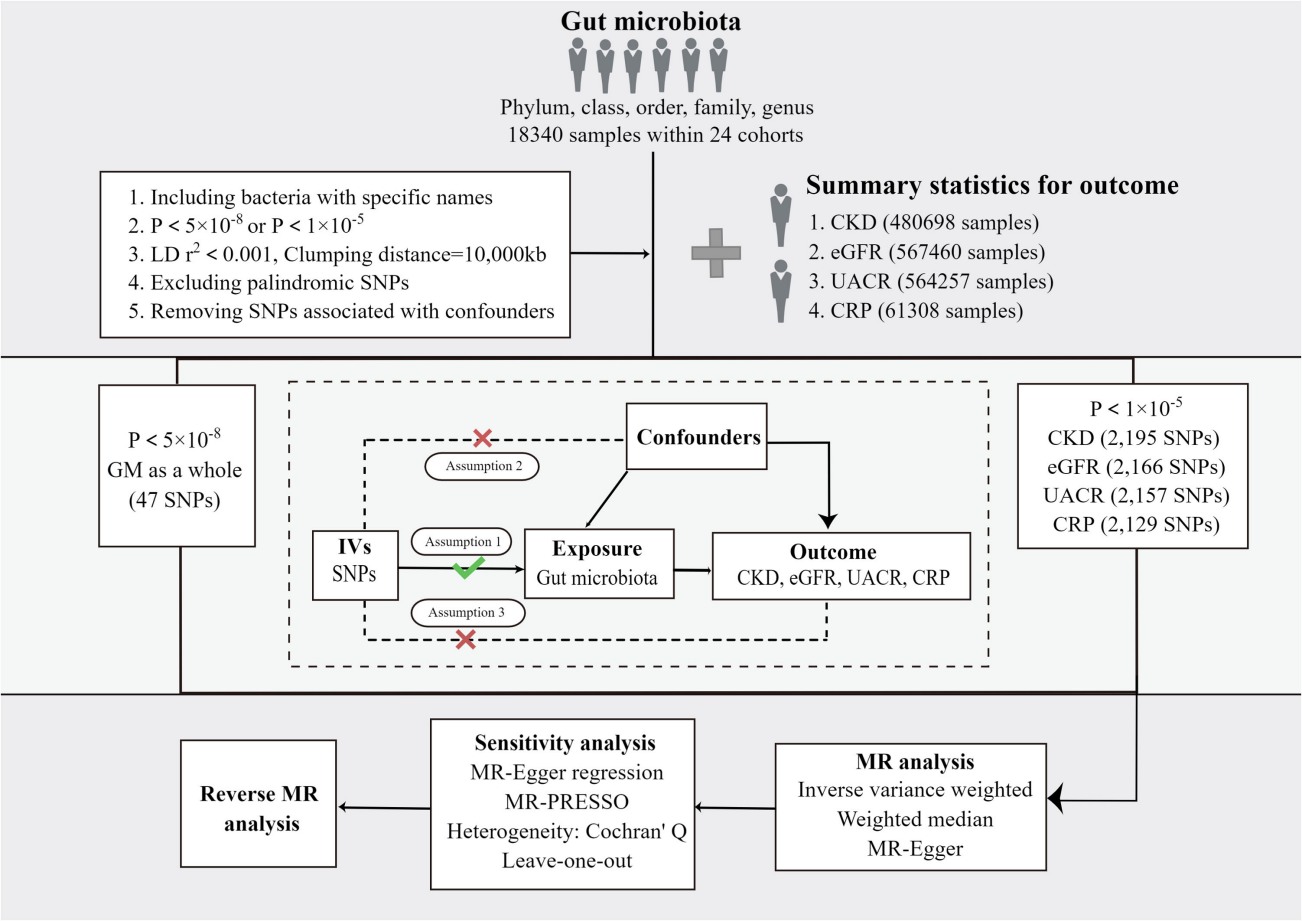
The study design and major assumptions of the Two-sample MR study. GM, gut microbiota; LD, linkage disequilibrium; SNP, single nucleotide polymorphism; IV: instruments variable; CKD, chronic kidney disease; eGFR, estimated glomerular filtration rate; UACR, urine albumin creatine ratio; CRP, c-reactive protein; MR-PRESSO, Mendelian Randomization Pleiotropy RESidual Sum and Outlier.

In this study, four sets of causal relationships were assessed by two-sample MR method: GM and CKD, GM and eGFR, GM and UACR, GM and CRP. The overall study flow is displayed in **Figure 1**. To ensure the reliability of MR results, three basic assumptions (21) must be conformed: (I) IVs significantly correlated with exposure are used in the analysis. (II) The IVs are independent of confounding factors affecting exposure and outcome. (III) The IVs are not horizontally pleiotropic, that is, the IVs have effects on outcome only through exposure.

### 2.2 Data sources of instruments variables

The largest GWAS meta-analysis of GM to date is from the MiBioGen consortium, containing 25 cohorts totaling 18,340 participants of European ancestry from 11 countries (22). From 211 bacterial taxa in total, this study ultimately identified 122,110 variant sites at 5 levels: phylum, class, order, family, and genus. To ensure the accuracy of the data, our study excluded 15 bacterial taxa of unknown family or genus, leaving 196.

Accurate measurement of renal function is difficult to achieve, so the use of biomarkers is necessary (23). Thus, we selected four sets of IVs to represent the renal function and chronic systemic inflammation. Among them, CKD is considered a chronic disease associated with impairment of renal function. eGFR is an important indicator of renal filtration function (24). UACR, a reflection of the degree of urinary protein, is the main clinical diagnostic criteria for CKD (25). CRP is one of the inflammatory markers measuring the severity of the inflammation status (26). The CKD, eGFR and UACR related summarized level GWAS data were collected from the CKDGen consortium’s meta-analysis of GWAS from participants of European ancestry. Wuttke et al. have reported CKDGen consortium in detail (27), so we did not elaborate here. With 480698 samples from 23 European ancestry cohorts, the GWAS meta-analysis of CKD comprised 41395 samples in the trial group and 439303 samples in the control group. GWAS meta-analysis of eGFR comprised 567460 European ancestry samples from 54 cohorts. The data of UCAR included 54 GWAS summary statistics of 564,257 participants. GWAS summary statistics related to CRP were obtained from The MRC IEU OpenGWAS data infrastructure (28). The dataset name is c-reactive protein (ID: ieu-b-4764) and it contains 8036590 single nucleotide polymorphism (SNP) in a sample size of 61308.

### 2.3 Selection of instruments variables

Quality checks of SNPs were performed to obtain eligible IVs: (I) GM related SNPs must reach a threshold (*P* < 5×10^-8^) with genome-wide significance. To obtain more comprehensive results, another set of SNPs reaching locus-wide significance level (*P* < 1×10^-5^) as IV was selected. (II) No linkage disequilibrium (LD) existed among GM-associated IVs, and a clumping process (r^2^ < 0.001, clumping distance = 10000kb) was performed on the screened SNPs to retain independent ones. (III) Moreover, for SNPs that were not available in GWASs of the outcome, we used the LD proxy search on the online platform (https://snipa.helmholtz-muenchen.de/snipa3/index.php/) to replace them with the proxy SNPs identified in high-LD (r2 > 0.8) or discard them if the proxies were not available (IV) The effects of GM-associated IVs on both exposure and outcome corresponded to the same alleles, so the palindromic SNPs were removed. (V) To avoid SNPs associated with potential risk factors for outcome, the PhenoScanner V2 website was used to retrieve these SNPs and exclude those associated with potential confounders or risk factors. (VI) To avoid bias caused by weak IVs, we calculated the intensity of IVs using F=R ^2^ (n-k-1)/k (1-R ^2^) (29, 30). R ^2^ represents the exposure variance explained by the selected SNPs, n is the sample size, and k represent the number of included instrumental variables. We excluded weak IVs with F < 10 (31).

### 2.4 Ethics statement

In this study, all the summary-level data were published available de-identified ones, which were authorized by the Ethical Standards Committee. No independent ethical approval was necessary during the research.

### 2.5 Mendelian randomization analysis

When no horizontal pleiotropy was available, the IVW method was used in this study as the primary method for inferring 4 sets of causal relationships: GM and CKD, GM and eGFR, GM and UACR, GM and CRP (32). To detect the presence of heterogeneity, we performed Cochran’ Q test. If there was significant heterogeneity (*P* < 0.05), a random-effects IVW model was adopted, otherwise a fixed-effects IVW model was applied (33). Moreover, to obtain more robust results under broader conditions, the weighted median (WM) approach and MR-Egger method were adopted to complement the IVW method. The additional methods need to satisfy the respective model assumptions: the WM approach assumes that at least half of the SNPs are free of pleiotropy (34). If the number of SNPs possessing pleiotropy exceeds 50%, the MR-Egger inference is still robust (35). In this study, the causality of exposure and outcome was considered to exist if the results of the main MR analysis reached a nominal significance (*P* < 0.05). The result would be regarded as significant and stable if it was supported by one or more additional methods simultaneously (36), and we would provide a focused discussion on such results.

To avoid the interference of pleiotropy to MR hypothesis, sensitivity analysis on the study results was performed: MR-Egger regression was used to estimate the potential horizontal pleiotropy of the included SNPs, and the results were considered to have horizontal pleiotropy if *P* < 0.05 (35). Considering the lower precision and statistical efficacy of MR-Egger regression, we used Mendelian randomization pleiotropy residual sum and outlier (MR-PRESSO) to examine any deviations to reflect pleiotropy bias and to give the causal effect of excluding outliers (37). The results of the sensitivity analysis for all IVs are presented in the **Supplementary Tables 1**, **2**. In addition, we conducted leave-one-out sensitivity analysis on significant results, in order to determine whether the significant causal association of the MR analysis was caused by a single IV (38).

### 2.6 Reverse-direction Mendelian randomization analysis

In MR analysis, additional reverse-direction MR analysis on stable and significant results was performed to test whether gene-predicted CKD, eGFR, UACR, and CRP would be causal to GM. The steps of reverse-direction MR analysis were the same as those of MR analysis.

## 3 Results

### 3.1 Selection of IVs related GM

**Figure 2:**
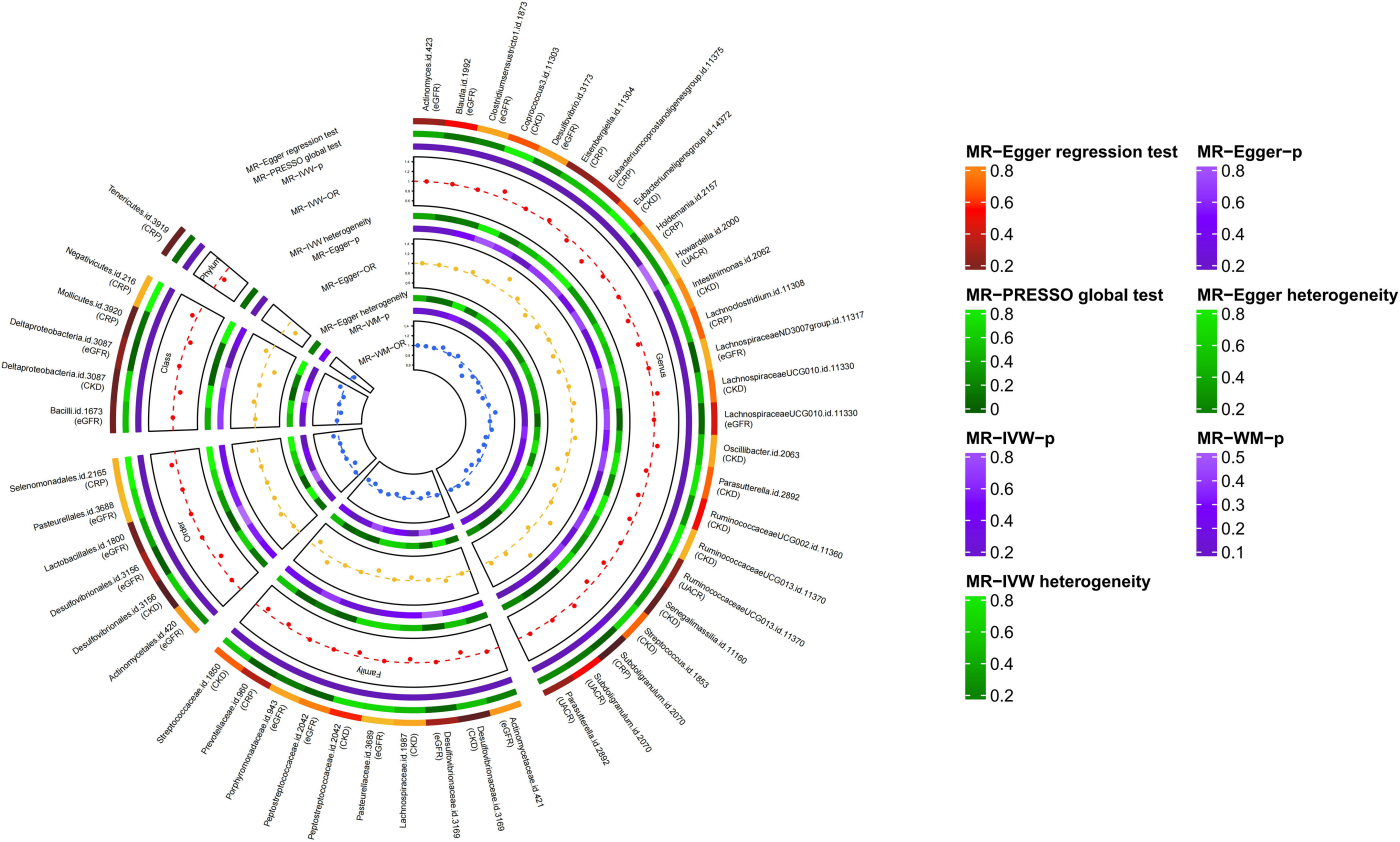
Results of MR study and sensitivity analysis between GM and CKD, GM and eGFR, GM and UACR, GM and CRP (locus-wide significance, *P*<1×10^-5^).

**Figure 3:**
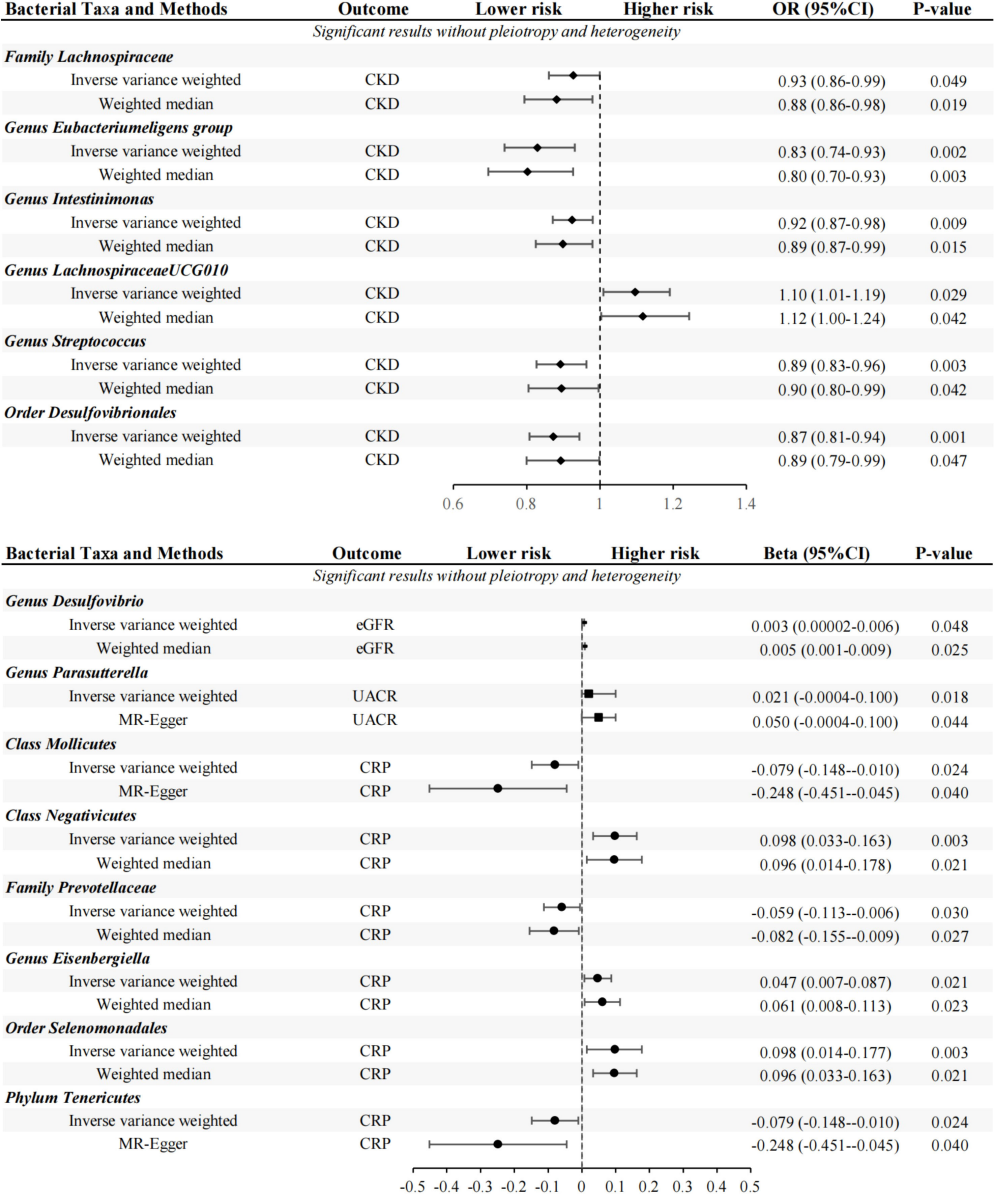
MR results of GM taxa with a significant causal relationships to CKD, EGFR, UACR and CRP (locus-wide significance, *P*<1×10^-5^).

Through a series of quality control measures, at the genome-wide significance level (*P* < 5×10^-8^), 12 SNPs, 11 SNPs, 12 SNPs and 12 SNPs were used for genetic prediction of CKD, eGFR, UACR and CRP, respectively. At the locus-wide significance level (*P* < 1×10^-5^), 2195 SNPs, 2166 SNPs, 2157 SNPs and 2129 SNPs were used for gene prediction of CKD, eGFR, UACR and CRP, respectively (**Supplementary Tables 3**, **4**). And the F-statistic values of the SNPs were all met (**Supplementary Tables 3**, **4**) (39). Based on the locus-wide significance level, we identified 16, 17, 4, and 10 bacterial taxa causally associated with CKD, eGFR, UACR, and CRP in the primary MR analysis, respectively, as detailed in **Supplementary Table 5** and **Figures 2**, **3**. After additional MR analysis and sensitivity analysis, only 6, 1, 1 and 6 bacterial taxa remained robust to the results of CKD, eGFR, UACR, and CRP, respectively (**Table 1**).

**Table 1:** Significant MR analysis results of causal links between GM and CKD, eGFR, UACR, CRP (*P*<1×10^-5^).

Human gut microbiota	Nsnps	Traits	Method	OR	OR (95% CI)	Beta	*P*-value	MR-Egger Regression		Heterogeneity (IVW)		Mean F
								Egger Intercept	*P*-value	Cochran’s Q	*P*-value	
Family *Lachnospiraceae*	17	CKD	WM	0.882	0.794-0.980	-0.126	0.019	-0.00063	0.923	15.430	0.493	21.040
			IVW	0.927	0.860-1.000	-0.075	0.049					
Genus *Eubacteriumeligens group*	7	CKD	WM	0.803	0.695-0.927	-0.22	0.003	-0.00802	0.702	1.434	0.964	20.734
			IVW	0.83	0.739-0.931	-0.187	0.002					
Genus *Intestinimonas*	17	CKD	WM	0.899	0.825-0.980	-0.107	0.015	0.00153	0.830	13.151	0.662	21.407
			IVW	0.924	0.871-0.980	-0.079	0.009					
Genus *LachnospiraceaeUCG010*	10	CKD	WM	1.117	1.004-1.244	0.111	0.042	0.00312	0.746	9.017	0.436	21.578
			IVW	1.096	1.010-1.190	0.092	0.029					
Genus *Streptococcus*	15	CKD	WM	0.895	0.805-0.996	-0.111	0.042	-0.00420	0.710	10.831	0.699	22.512
			IVW	0.892	0.827-0.963	-0.114	0.003					
Order *Desulfovibrionales*	12	CKD	WM	0.894	0.800-0.999	-0.113	0.047	-0.01546	0.066	8.528	0.665	21.488
			IVW	0.873	0.808-0.944	-0.136	0.001					
Genus *Desulfovibrio*	10	eGFR	WM	1.005	1.001-1.009	0.005	0.025	0.00007	0.894	12.589	0.182	21.674
			IVW	1.003	1.000-1.007	0.003	0.048					
Class *Mollicutes*	11	CRP	MR-egger	0.78	0.637-0.956	-0.248	0.04	0.01580	0.120	16.648	0.083	21.298
			IVW	0.924	0.862-0.990	-0.079	0.024					
Class *Negativicutes*	11	CRP	WM	1.101	1.014-1.194	0.096	0.021	-0.00032	0.962	2.713	0.987	21.709
			IVW	1.103	1.034-1.177	0.098	0.003					
Family *Prevotellaceae*	15	CRP	WM	0.921	0.856-0.991	-0.082	0.027	-0.01305	0.063	15.349	0.355	22.150
			IVW	0.943	0.894-0.994	-0.059	0.03					
Genus *Eisenbergiella*	10	CRP	WM	1.063	1.008-1.120	0.061	0.023	-0.02104	0.229	8.352	0.499	21.278
			IVW	1.048	1.007-1.091	0.047	0.021					
Order *Selenomonadales*	11	CRP	WM	1.101	1.015-1.194	0.096	0.021	-0.00032	0.962	2.713	0.987	21.709
			IVW	1.103	1.034-1.177	0.098	0.003					
Phylum *Tenericutes*	11	CRP	MR-egger	0.78	0.637-0.956	-0.248	0.04	0.01580	0.120	16.648	0.083	21.298
			IVW	0.924	0.862-0.990	-0.079	0.024					
Genus *Parasutterella*	15	UACR	MR-egger	1.051	1.000-1.105	0.05	0.044	-0.00287	0.007	9.032	0.770	22.369
			IVW	1.021	1.004-1.039	0.021	0.018					

Nsnps, number of single nucleotide polymorphisms; OR, odds ratio; CKD, chronic kidney disease; eGFR, estimated glomerular filtration rate; CRP, c-reactive protein; UACR, urine albumin creatine ratio; IVW, inverse variance weighted method; WM, weighted median method.

### 3.2 Locus-wide significance threshold *P*<1×10^-5^

#### 3.2.1 CKD

Primary MR analysis showed that 16 bacterial taxa were associated with CKD risk (**Supplementary Table 5**; **Figure 2**). However, only 6 bacterial taxa remained robust in subsequent additional and sensitivity analysis (**Table 1**). Precisely, higher genetically predicted *Lachnospiraceae*, *Eubacteriumeligens* group, *Intestinimonas*, *Streptococcus*, and *Desulfovibrionales* were related to a lower risk of CKD [odds ratio (OR): 0.927, 95% confidence interval (CI): 0.79-0.98, *P*=0.049 for *Lachnospiraceae*; and OR: 0.830, 95% CI: 0.74-0.93, *P*=0.002 for *Eubacteriumeligens* group, and OR: 0.924, 95% CI: 0.87-0.98, *P*=0.009 for *Intestinimonas*, and OR: 0.892, 95% CI: 0.82-0.96, *P*=0.003 for *Streptococcus*, and OR: 0.873, 95% CI: 0.81-0.94, *P*=0.001 for *Desulfovibrionales*]. Conversely, higher genetic prediction of *LachnospiraceaeUCG010* was related to a higher risk of CKD [OR: 1.096, 95% CI: 1.01-1.19, *P*=0.029].

#### 3.2.2 eGFR

Seventeen bacterial taxa associated with eGFR were identified from primary MR analysis (**Supplementary Table 5**; **Figure 2**). After additional and sensitivity analysis, only the results for *Desulfovibrio* remained robust, as detailed in **Table 1**. Results from the IVW method showed that higher abundance of *Desulfovibrio* was related to higher eGFR [beta: 0.003, 95% CI: 2.37×10^-5^-6.48×10^-3^, *P*=0.048], and this result was also supported by the WM approach [beta: 0.005, 95% CI: 5.79×10^-4^-8.51×10^-3^, *P*=0.025], suggesting a protective effect of *Desulfovibrio* on eGFR decline.

#### 3.2.3 UACR

Primary MR analysis confirmed that 4 bacterial taxa were associated with UACR (**Supplementary Table 5**; **Figure 2**). Sensitivity analysis and additional analysis only supported the results for *Parasutterella* (**Table 1**). IVW results showed that higher abundance of *Parasutterella* was a risk factor for proteinuria and was related to higher levels of UACR [beta: 0.021, 95% CI: 0.01-0.04, *P*=0.018].

#### 3.2.4 CRP

Ten bacterial taxa were proved to be causally related to CRP in the primary MR analysis (**Supplementary Table 5**; **Figure 2**). However, sensitivity and additional analysis supported the results for only 6 bacterial taxa, as shown in **Table 1**. MR analysis of IVW showed that genetically predicted *Mollicutes* [beta: -0.079, 95% CI: -0.15–0.01, *P*=0.024], *Prevotellaceae* [beta: -0.059, 95% CI: -0.11–0.01, *P*=0.030] and *Tenericutes* [beta: -0.079, 95% CI: -0.15–0.01, *P*=0.024] were associated with lower CRP levels and were protective factors against inflammation. Conversely, genetically predicted *Negativicutes* [beta: 0.098, 95% CI: 0.03–0.16, P=0.003], *Eisenbergiella* [beta: 0.047, 95% CI: 0.01–0.09, *P*=0.021], and *Selenomonadales* [beta: 0.098, 95%CI: 0.03–0.16, *P*=0.003] were associated with higher CRP levels (**Table 1**).

As shown in **Supplementary Table 6**, the intercepts of the MR-Egger regressions all did not deviate from the null, suggesting no evidence for horizontal pleiotropy (*P* > 0.05 for all intercepts). We further validated the MR-Egger regression results using MR-PRESSO, and no evidence of outliers was found (**Supplementary Table 5**). Leave-one-out analysis found no outliers as in MR-PRESSO (**Supplementary Figures 1A–N**). Without heterogeneity and pleiotropy, outcomes of the above MR analysis were reliable and robust.

### 3.3 Genome-wide significance threshold *P*<5×10^-8^

At this stage, we analyzed gut microbiota at five levels (phylum, class, order, family, and genus) based on SNPs available in the gut microbiome GWAS summary data. The results showed no significant causal relationship with CKD, eGFR, UACR or CRP when MR analysis was conducted on each taxonomic level (**Table 2**). In sensitivity analysis, no intercepts of MR-Egger regression were diverged from the null value, suggesting the absence of evidence for horizontal pleiotropy (P > 0.05). MR-PRESSO results showed outliers in sensitivity analysis of UACR at the genus level, then we removed rs35866622, rs602075 and re-analyzed the results, the results did not change significantly (p=0.1095194, beta=0.0125, OR=1.0126). The results of MR analysis could not prove a significant causal relationship between GM and CKD, eGFR, UACR or CRP, because there were too few eligible IVs (**Supplementary Table 2**).

**Table 2:** MR analysis results of causal links between GM and CKD, eGFR, UACR, CRP (*P*<5×10^-8^).

Human gut microbiota	Nsnps	Traits	Method	OR	OR (95%)	Beta	*P*-value	MR-Egger Regression		Heterogeneity (IVW)		F
								Egger Intercept	*P*-value	Cochran’s Q	*P*-value	
Level of class	1	CKD	Wald ratio	0.973	0.795-1.190	-2.781E-02	0.787	NA	NA	NA	NA	85.376
Level of family	6	CKD	MR-Egger	1.507	0.915-2.482	4.101E-01	0.183	-0.054	0.159	3.277	0.657	40.243
Level of family	6	CKD	WM	0.987	0.895-1.088	-1.342E-02	0.789					
Level of family	6	CKD	IVW	0.976	0.903-1.055	-2.419E-02	0.542					
Level of genus	12	CKD	MR-Egger	0.881	0.709-1.093	-1.271E-01	0.276	0.012	0.318	15.089	0.178	35.942
Level of genus	12	CKD	WM	0.967	0.902-1.038	-3.313E-02	0.354					
Level of genus	12	CKD	IVW	0.984	0.923-1.048	-1.640E-02	0.613					
Level of order	3	CKD	MR-Egger	1.344	0.683-2.645	2.955E-01	0.550	-0.039	0.539	0.807	0.668	48.587
Level of order	3	CKD	WM	0.990	0.869-1.128	-1.036E-02	0.876					
Level of order	3	CKD	IVW	0.994	0.891-1.109	-5.723E-03	0.918					
Level of phylum	1	CKD	Wald ratio	0.983	0.765-1.263	-1.731E-02	0.893	NA	NA	NA	NA	58.164
Level of class	1	eGFR	Wald ratio	1.004	0.997-0.011	3.588E-03	0.317	NA	NA	NA	NA	85.376
Level of family	6	eGFR	MR-Egger	0.997	0.978-0.017	-2.835E-03	0.793	0.000	0.800	4.365	0.498	40.243
Level of family	6	eGFR	WM	1.000	0.997-1.003	2.133E-04	0.909					
Level of family	6	eGFR	IVW	1.000	0.998-1.003	-1.305E-04	0.928					
Level of genus	12	eGFR	MR-Egger	1.055	0.984-1.131	5.373E-02	0.123	-0.001	0.101	17.306	0.099	35.942
Level of genus	12	eGFR	WM	1.015	0.995-1.035	1.442E-02	0.532					
Level of genus	12	eGFR	IVW	1.013	0.997-1.028	1.248E-02	0.887					
Level of order	3	eGFR	MR-Egger	1.002	0.978-1.028	2.212E-03	0.890	0.000	0.991	0.369	0.831	48.587
Level of order	3	eGFR	WM	1.002	0.998-1.007	2.165E-03	0.346					
Level of order	3	eGFR	IVW	1.002	0.998-1.006	2.041E-03	0.320					
Level of phylum	1	eGFR	Wald ratio	1.005	0.996-1.014	4.616E-03	0.317	NA	NA	NA	NA	58.164
Level of class	1	UACR	Wald ratio	1.036	0.995-1.078	3.498E-02	0.090	NA	NA	NA	NA	85.376
Level of family	6	UACR	MR-Egger	0.995	0.887-0.118	-4.601E-03	0.942	0.002	0.752	2.325	0.803	40.243
Level of family	6	UACR	WM	1.013	0.989-1.037	1.245E-02	0.308					
Level of family	6	UACR	IVW	1.015	0.996-1.034	1.510E-02	0.114					
Level of genus	12	UACR	MR-Egger	0.935	0.860-1.016	-6.714E-02	0.145	0.009	0.070	52.240	0.000	35.942
Level of genus	12	UACR	WM	1.010	0.991-1.028	9.548E-03	0.306					
Level of genus	12	UACR	IVW (random)	1.015	0.987-1.044	1.491E-02	0.302					
Level of order	3	UACR	MR-Egger	0.960	0.814-1.131	-4.129E-02	0.709	0.008	0.588	0.735	0.692	48.587
Level of order	3	UACR	WM	1.020	0.990-1.051	1.991E-02	0.195					
Level of order	3	UACR	IVW	1.022	0.996-1.048	2.132E-02	0.105					
Level of phylum	1	UACR	Wald ratio	1.044	0.993-1.097	4.270E-02	0.093	NA	NA	NA	NA	58.164
Level of class	1	CRP	Wald ratio	0.883	0.782-0.997	-1.247E-01	0.044	NA	NA	NA	NA	85.376
Level of family	6	CRP	MR-Egger	1.166	0.839-1.620	1.537E-01	0.412	-0.026	0.276	3.645	0.602	40.243
Level of family	6	CRP	WM	0.939	0.875-1.007	-6.334E-02	0.079					
Level of family	6	CRP	IVW	0.946	0.896-0.999	-5.505E-02	0.046					
Level of genus	12	CRP	MR-Egger	1.051	0.913-1.210	5.020E-02	0.499	-0.010	0.218	15.913	0.144	36.263
Level of genus	12	CRP	WM	0.972	0.926-1.022	-2.799E-02	0.266					
Level of genus	12	CRP	IVW	0.961	0.920-1.004	-3.969E-02	0.075					
Level of order	3	CRP	MR-Egger	1.455	0.918-2.307	3.753E-01	0.356	-0.056	0.318	3.592	0.166	48.587
Level of order	3	CRP	WM	0.920	0.837-1.011	-8.324E-02	0.083					
Level of order	3	CRP	IVW	0.952	0.863-1.050	-4.958E-02	0.323					
Level of phylum	1	CRP	Wald ratio	0.907	0.779-1.055	-9.809E-02	0.205	NA	NA	NA	NA	58.164

Nsnps, number of single nucleotide polymorphism; OR, odds ratio; CKD, chronic kidney disease; eGFR, estimated glomerular filtration rate; CRP, c-reactive protein; UACR, urine albumin creatine ratio; IVW, inverse variance weighted method; WM, weighted median method; NA, Not Applicable.

### 3.4 Reverse-direction Mendelian randomization analysis

Finally, reverse MR analysis was performed for the 14 bacterial taxa presenting significant results with CKD, eGFR, UACR and CRP. After additional analysis and sensitivity analysis, no significant and stable results were found. The specific information is presented in **Supplementary Table 7**.

## 4 Discussion

This research was not the first one exploring the cause-and-effect relationship between GM and CKD. However, the innovative points of this article remedied the shortcomings of the past studies: (I) GM data used here was the latest large GWAS dataset, which contains 196 bacterial taxa at 5 levels, consisting of 9 phyla, 16 classes, 20 orders, 32 families, 119 genera, in contrast, previous studies only estimated the effect of 8 microbiota genera on CKD (40). The bacterial taxa we studied in this research included but were not limited to those analyzed in previous studies. (II) The selection of IVs was more rigorous, and two thresholds were used to select independent SNPs. In contrast, previous studies did not select SNPs strongly associated with exposure and did not perform the aggregation process, which might seriously affect the reliability and robustness of MR analysis results. (III) Chronic systemic inflammation in CKD is a strong predictor of CKD prognosis, and UACR is sensitive and specific to the overall renal injury profile. Hence, this study revealed not only the causal relationship between GM and CKD, eGFR, but also GM and UACR, CRP. In conclusion, our findings expanded the bacterial taxa associated with CKD and revealed that GM played a regulatory role in CKD and its inflammation state.

**Figure 4:**
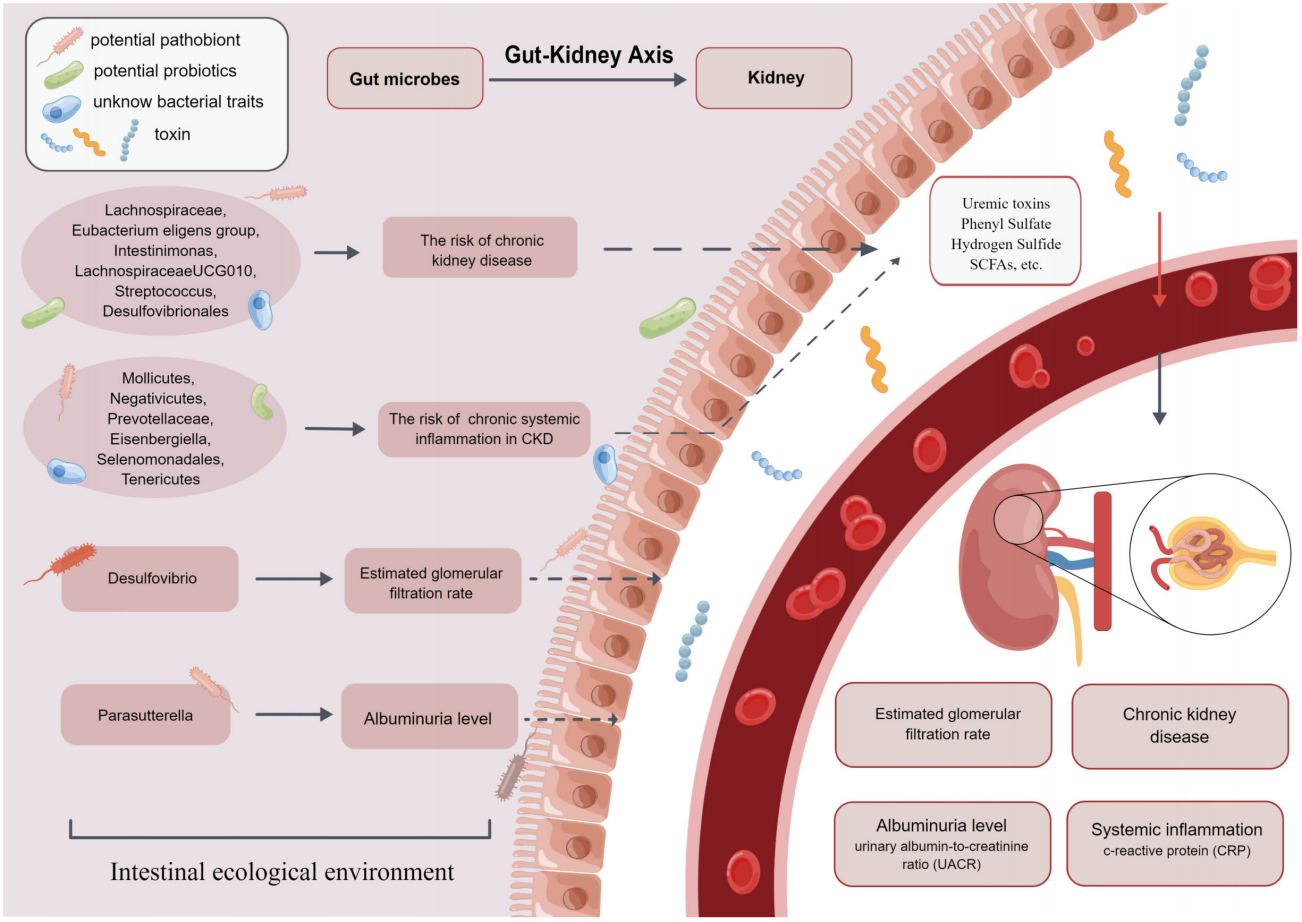
The present MR study reveals that GM causally influences CKD, eGFR, UACR and CRP, which supports the existence of gut-kidney axis.

The human intestine contains more than 1,000 bacterial taxa, 10^14^ in total, which are involved in the metabolic processes of substances in the body and regulate immune function. Several studies (41, 42) found that the composition and abundance of GM were different between healthy individuals and CKD patients. The diversity and different abundance of bacterial taxa may contribute to the development of CKD and CKD co-morbidities such as chronic systemic inflammation. Therefore, the causal relationships between GM and CKD, as well as GM and chronic systemic inflammation in CKD, were explored in this study. After additional analysis and sensitivity analysis, 14 bacterial taxa had a significant causal relationship with our outcome at the phylum, class, order, family, and genus levels (**Figure 4**).

Family *Lachnospiraceae* isa critical member of GM. Previous studies demonstrated that *Lachnospiraceae* took an essential part in the pathogenesis of CKD (43, 44), however, there were conflicting findings among these studies. Lai et al. (45) found that the abundance of *Lachnospiraceae* was increased in mice with kidney damage and in patients with CKD, which might predict that it was a risk bacterial taxon for CKD. Conversely, other observational studies (44) showed that the abundance of *Lachnospiraceae* in stool samples from patients with CKD was reduced. Moreover, the abundance of *Lachnospiraceae* was negatively correlated with serum creatinine, urea nitrogen and other indicators of renal function, and our study supported these findings. The reason may be that *Lachnospiraceae* is the primary producer of short-chain fatty acids (SCFAs) (43). SCFAs act as a link between GM and within-host environments. They play essential roles in correcting pH value and ensuring the integrity of the intestinal epithelium (46). SCFAs also induce renin secretion and the development of renal cell subtypes to control blood pressure, thereby delaying the progression of CKD (47).

Furthermore, a study by Hu et al. (48) found an increased abundance of genus *Intestinimonas* in CKD patients, suggesting that it might be a risk factor for CKD. The mechanism may be the involvement of genus *Intestinimonas* in the production of tryptophan-derived uremic toxins, which damage the renal vasculature and the circulatory system (49). However, these findings are inconsistent with our conclusions. It is possible that gene-gene interactions and gene-environment interactions result in the same changes in GM but cause different results. In addition, these observational studies may draw biased conclusions because of their smaller sample size (12 rats) and shorter trial duration (2 weeks). In addition to the bacteria mentioned above that are causally related to CKD, in the present study, genus *Eubacterium eligens* group, genus *Streptococcus*, and order *Desulfovibrionales*, all showed a positive effect on CKD, whereas genus *LachnospiraceaeUCG010* indicated a negative effect on CKD.

Two-sample MR analysis also found that higher abundance of genus *Desulfovibrio* was positively associated with higher levels of eGFR. An observational study (50) supporting our view confirmed that the abundance of order *Desulfovibrionales* was low in stool samples from patients presenting with eGFR <15 mL/min/1.73 m^2^, implying that order *Desulfovibrionales* was a beneficial bacterial taxon for eGFR. Genus *Desulfovibrio* is a bacterial genera pattern of order *Desulfovibrionales*, which may be relevant to the findings of this study. However, Zhao et al. (51) suggested that the abundance of genus *Desulfovibrio* was higher in patients with CKD whose eGFR was significantly reduced. Although the direction of association between genus *Desulfovibrio* and eGFR varied considerably in different studies, the current consensus concluded that genus *Desulfovibrio* could metabolize renoprotective SCFAs (14). It also produces more hydrogen sulfide, causing metabolic impairment and toxicity in the kidney (52, 53), which consequently affects eGFR.

In the MR analysis of GM and proteinuria, our results showed that genus *Parasutterella* was positively associated with higher UACR. This finding is also supported by previous studies: Feng et al. (54) found that GM disorders such as genus *Parasutterella* increased in abundance was positively correlated with increased proteinuria in CKD model rats. The mechanism may involve phenyl sulfate (PS) (17), an enteric-derived uremic toxin (55) that can increase proteinuria by damaging podocytes (56). The level of PS is found to be significantly associated with UACR in microalbuminuric patients. It is the only element that predicts the progression of UACR in microalbuminuric patients over the next two years (57). A few bacterial taxa such as *Parasutterella* and *Citrobacter* contain TPL, which synthesizes tyrosine into phenol, which is further metabolized to PS (58). Therefore, GM intervention will constitute a new approach towards the prevention and treatment of proteinuria.

Researchers have long worked on the relationship between GM and chronic systemic inflammation in CKD. One of the common comorbidities of CKD is chronic systemic inflammation. To some extent, chronic inflammation can reflect the renal function status of CKD patients, and it is predictive of CKD prognosis and related comorbidities (7, 59). Previous study (60) suggested that the amount and abundance of GM producing SCFAs were increased, whereas GM producing uremic toxins were increased in CKD patients. Toxins disrupted intestinal tight junctions, penetrated from the damaged intestinal mucosa into the blood, and produced systemic chronic inflammation (60). The regulation of imbalanced GM has become an increasingly popular topic in correcting chronic inflammation. It has been considered (61) that regulating GM abundance by supplementation with probiotics and prebiotics is a proven prevention and treatment method to modify the inflammatory status of CKD patients effectively. Our findings indirectly confirmed that differences in GM abundance do have an effect on inflammatory status.

In the present study, high abundance of family *Prevotellaceae*, class *Mollicutes*, and phylum *Tenericutes* were negatively correlated with CRP. Vaziri et al. (62) found that in stool samples from ESRD patients, family *Prevotellaceae* abundance decreased, leading to increased levels of inflammatory factors (such as CRP) in patients. This was consistent with some of our findings. Family *Prevotellaceae* is the main bacterial taxon producing SCFAs. SCFAs play an important role in maintaining intestinal epithelial integrity and activating FFAR2 to suppress inflammation (63). Li et al. (64) found that patients with CKD4-5 had improved inflammatory status after appropriate intake of SCFAs. Therefore, further study of GM such as family *Prevotellaceae*, class *Mollicutes* and phylum *Tenericutes* may provide theoretical support for the therapeutic strategy to improve chronic systemic inflammation in CKD by intervening GM. In addition to the mentioned GM above are associated with chronic systemic inflammation in CKD, our results also showed that high abundance of class *Negativicutes*, genus *Eisenbergiella*, and order *Selenomonadales* were positively associated with CRP levels.

The mechanisms of GM effects on chronic systemic inflammation in CKD have been proposed so far. The main mechanisms focus on the impact of intestinal endotoxins and GM metabolites (e.g., IS, PCS) (65) on inflammatory responses. This includes the induction of renal tubular oxidative stress and inflammatory responses by IS and PCS (65), and the induction of cytokine secretion by renal interstitial cells to promote inflammatory responses (66). However, no human or rodent model studies have yet explored class *Negativicutes*, genus *Eisenbergiella*, and order *Selenomonadales*, so researches for these bacteria taxa may become an approach to discovering novel markers for predicting chronic inflammation in CKD.

This study has certain advantages and limitations. First, the advantage is that MR analysis was used, which is a novel genetic statistical method to detect causality. It can avoid confounders and reverse causality. Limitations include (I) GM data were only classified above the genus level. Thus, the causality of CKD and chronic systemic inflammation in CKD could not be concluded at species, strain, or more specialized levels. (II) Since the GWAS data only included European ancestry, the conclusions of this research could not be extended to cover other races. (III) Considering the multi-stage statistics process and biological plausibility, stringent multiple testing calibration may overlook the potential GM with CKD and chronic systemic inflammation in CKD. Hence, we did not strictly follow the multi-corrected p-values to screen GM. (IV) Classes are subcategories of phyla. So the SNP data contained in phylum, class, order, family, and genus may have significant overlap, which may lead to the reproducibility of MR analysis results.

## 5 Conclusion

Four sets of causal relationships were confirmed in this work: GM and CKD, GM and eGFR, GM and UACR, GM and CRP; however, the reverse causality was not identified. In addition, the present study identified specific bacterial taxa associated with CKD, eGFR, UACR, and CRP, respectively, which might be novel biomarkers for further research of CKD and chronic systemic inflammation in CKD. The study of these bacterial taxa may contribute to the prevention and treatment of CKD and chronic systemic inflammation in CKD, and provide a theoretical basis for studying the mechanism governing gut-kidney axis.

## Data availability statement

The original contributions presented in the study are included in the article/**Supplementary Files**, further inquiries can be directed to the corresponding author.

## Author contributions

FR: Formal Analysis, Writing – original draft. QiuJ: Formal Analysis, Writing – review and editing. QiJ: Formal Analysis, Writing – review and editing. YQ: Data curation, Formal Analysis, Writing – review and editing. XR: Data curation, Writing – review and editing. TL: Writing – review and editing. YZ: Conceptualization, Funding acquisition, Investigation, Writing – review and editing.

## Supplementary material

Supplementary Figure 1
**(A)** The result of sensitivity analysis of the causal effect of family *Lachnospiraceae* on CKD risk. **(B)** The result of sensitivity analysis of the causal effect of genus *Eubacteriumeligens* group on CKD risk. **(C)** The result of sensitivity analysis of the causal effect of genus *Intestinimonas* on CKD risk. **(D)** The result of sensitivity analysis of the causal effect of genus *LachnospiraceaeUCG010* on CKD risk. **(E)** The result of sensitivity analysis of the causal effect of genus *Streptococcus* on CKD risk. **(F)** The result of sensitivity analysis of the causal effect of genus *Desulfovibrionales* on CKD risk. **(G)** The result of sensitivity analysis of the causal effect of order *Desulfovibrio* on eGFR. **(H)** The result of sensitivity analysis of the causal effect of class *Mollicutes* on CRP. **(I)** The result of sensitivity analysis of the causal effect of class *Negativicutes* on CRP. **(J)** The result of sensitivity analysis of the causal effect of family *Prevotellaceae* on CRP. **(K)** The result of sensitivity analysis of the causal effect of genus *Eisenbergiella* on CRP. **(L)** The result of sensitivity analysis of the causal effect of order *Selenomonadales* on CRP. **(M)** The result of sensitivity analysis of the causal effect of phylum *Tenericutes* onCRP. **(N)** The result of sensitivity analysis of the causal effect of genus *Parasutterella* on UACR.

## References

[1] Lameire NH Levin A Kellum JA Cheung M Jadoul M Winkelmayer WC. Harmonizing acute and chronic kidney disease definition and classification: report of a Kidney Disease: Improving Global Outcomes (KDIGO) Consensus Conference. Kidney Int (2021) 100:516–26. doi: 10.1016/j.kint.2021.06.028 34252450

[2] Köttgen A Pattaro C Böger CA Fuchsberger C Olden M Glazer NL. New loci associated with kidney function and chronic kidney disease. Nat Genet (2010) 42:376–84. doi: 10.1038/ng.568 PMC2997674 20383146

[3] Böger CA Chen MH Tin A Olden M Köttgen A de Boer IH. CUBN is a gene locus for albuminuria. J Am Soc Nephrol (2011) 22:555–70. doi: 10.1681/asn.2010060598 PMC3060449 21355061

[4] Lv JC Zhang LX. Prevalence and disease burden of chronic kidney disease. Adv Exp Med Biol (2019) 1165:3–15. doi: 10.1007/978-981-13-8871-2_1 31399958

[5] Glassock RJ Warnock DG Delanaye P. The global burden of chronic kidney disease: estimates, variability and pitfalls. Nat Rev Nephrol (2017) 13:104–14. doi: 10.1038/nrneph.2016.163 27941934

[6] Akchurin OM Kaskel F. Update on inflammation in chronic kidney disease. Blood Purif (2015) 39:84–92. doi: 10.1159/000368940 25662331

[7] Bazeley J Bieber B Li Y Morgenstern H de Sequera P Combe C. C-reactive protein and prediction of 1-year mortality in prevalent hemodialysis patients. Clin J Am Soc Nephrol (2011) 6:2452–61. doi: 10.2215/cjn.00710111 PMC3186454 21868617

[8] Navaneethan SD Zoungas S Caramori ML Chan JCN Heerspink HJL Hurst C. Diabetes management in chronic kidney disease: synopsis of the 2020 KDIGO clinical practice guideline. Ann Intern Med (2021) 174:385–94. doi: 10.7326/m20-5938 33166222

[9] Kim HW Park JT Joo YS Kang SC Lee JY Lee S. Systolic blood pressure and chronic kidney disease progression in patients with primary glomerular disease. J Nephrol (2021) 34:1057–67. doi: 10.1007/s40620-020-00930-x 33555575

[10] Hsu CN Tain YL. Developmental origins of kidney disease: why oxidative stress matters? Antioxidants (Basel) (2020) 10:33. doi: 10.3390/antiox10010033 33396856 PMC7823649

[11] Carrero JJ Yilmaz MI Lindholm B Stenvinkel P. Cytokine dysregulation in chronic kidney disease: how can we treat it? Blood Purif (2008) 26:291–9. doi: 10.1159/000126926 18421214

[12] Sabatino A Regolisti G Brusasco I Cabassi A Morabito S Fiaccadori E. Alterations of intestinal barrier and microbiota in chronic kidney disease. Nephrol Dial Transplant (2015) 30:924–33. doi: 10.1093/ndt/gfu287 25190600

[13] Yang T Richards EM Pepine CJ Raizada MK. The gut microbiota and the brain-gut-kidney axis in hypertension and chronic kidney disease. Nat Rev Nephrol (2018) 14:442–56. doi: 10.1038/s41581-018-0018-2 PMC6385605 29760448

[14] Li F Wang M Wang J Li R Zhang Y. Alterations to the gut microbiota and their correlation with inflammatory factors in chronic kidney disease. Front Cell Infect Microbiol (2019) 9:206. doi: 10.3389/fcimb.2019.00206 31245306 PMC6581668

[15] Meijers BK Evenepoel P. The gut-kidney axis: indoxyl sulfate, p-cresyl sulfate and CKD progression. Nephrol Dial Transplant (2011) 26:759–61. doi: 10.1093/ndt/gfq818 21343587

[16] Lau WL Kalantar-Zadeh K Vaziri ND. The gut as a source of inflammation in chronic kidney disease. Nephron (2015) 130:92–8. doi: 10.1159/000381990 PMC4485546 25967288

[17] Kikuchi K Saigusa D Kanemitsu Y Matsumoto Y Thanai P Suzuki N. Gut microbiome-derived phenyl sulfate contributes to albuminuria in diabetic kidney disease. Nat Commun (2019) 10:1835. doi: 10.1038/s41467-019-09735-4 31015435 PMC6478834

[18] Tao S Tao S Cheng Y Liu J Ma L Fu P. Effects of probiotic supplements on the progression of chronic kidney disease: A meta-analysis. Nephrol (Carlton) (2019) 24:1122–30. doi: 10.1111/nep.13549 30561114

[19] Smith GD Ebrahim S. 'Mendelian randomization': can genetic epidemiology contribute to understanding environmental determinants of disease? Int J Epidemiol (2003) 32:1–22. doi: 10.1093/ije/dyg070 12689998

[20] Emdin CA Khera AV Kathiresan S. Mendelian randomization. Jama (2017) 318:1925–26. doi: 10.1001/jama.2017.17219 29164242

[21] Davey Smith G Hemani G. Mendelian randomization: genetic anchors for causal inference in epidemiological studies. Hum Mol Genet (2014) 23:R89–98. doi: 10.1093/hmg/ddu328 PMC4170722 25064373

[22] Kurilshikov A Medina-Gomez C Bacigalupe R Radjabzadeh D Wang J Demirkan A. Large-scale association analyses identify host factors influencing human gut microbiome composition. Nat Genet (2021) 53:156–65. doi: 10.1038/s41588-020-00763-1 PMC8515199 33462485

[23] Levey AS Inker LA. GFR as the "Gold standard": estimated, measured, and true. Am J Kidney Dis (2016) 67:9–12. doi: 10.1053/j.ajkd.2015.09.014 26708193

[24] Marini S Georgakis MK Chung J Henry JQA Dichgans M Rosand J. Genetic overlap and causal inferences between kidney function and cerebrovascular disease. Neurology (2020) 94:e2581–e91. doi: 10.1212/wnl.0000000000009642 PMC7455337 32439819

[25] Filippatos G Anker SD Agarwal R Ruilope LM Rossing P Bakris GL. Finerenone reduces risk of incident heart failure in patients with chronic kidney disease and type 2 diabetes: analyses from the FIGARO-DKD trial. Circulation (2022) 145:437–47. doi: 10.1161/circulationaha.121.057983 PMC8812430 34775784

[26] Carlsson AC Juhlin CC Larsson TE Larsson A Ingelsson E Sundström J. Soluble tumor necrosis factor receptor 1 (sTNFR1) is associated with increased total mortality due to cancer and cardiovascular causes - findings from two community based cohorts of elderly. Atherosclerosis (2014) 237:236–42. doi: 10.1016/j.atherosclerosis.2014.09.005 25255422

[27] Wuttke M Li Y Li M Sieber KB Feitosa MF Gorski M. A catalog of genetic loci associated with kidney function from analyses of a million individuals. Nat Genet (2019) 51:957–72. doi: 10.1038/s41588-019-0407-x PMC6698888 31152163

[28] Mitchell R Elsworth BL Raistrick CA Paternoster L Hemani G Gaunt TR. MRC IEU UK Biobank GWAS pipeline version 2. University of Bristol. (2019). doi: 10.5523/bris.pnoat8cxo0u52p6ynfaekeigi.

[29] Lawlor DA Harbord RM Sterne JA Timpson N Davey Smith G. Mendelian randomization: using genes as instruments for making causal inferences in epidemiology. Stat Med (2008) 27:1133–63. doi: 10.1002/sim.3034 17886233

[30] Mensah-Kane J Schmidt AF Hingorani AD Finan C Chen Y van Duijvenboden S. No clinically relevant effect of heart rate increase and heart rate recovery during exercise on cardiovascular disease: A mendelian randomization analysis. Front Genet (2021) 12:569323. doi: 10.3389/fgene.2021.569323 33679875 PMC7931909

[31] Burgess S Thompson SG. Avoiding bias from weak instruments in Mendelian randomization studies. Int J Epidemiol (2011) 40:755–64. doi: 10.1093/ije/dyr036 21414999

[32] Burgess S Butterworth A Thompson SG. Mendelian randomization analysis with multiple genetic variants using summarized data. Genet Epidemiol (2013) 37:658–65. doi: 10.1002/gepi.21758 PMC4377079 24114802

[33] Greco MF Minelli C Sheehan NA Thompson JR. Detecting pleiotropy in Mendelian randomisation studies with summary data and a continuous outcome. Stat Med (2015) 34:2926–40. doi: 10.1002/sim.6522 25950993

[34] Hartwig FP Davey Smith G Bowden J. Robust inference in summary data Mendelian randomization via the zero modal pleiotropy assumption. Int J Epidemiol (2017) 46:1985–98. doi: 10.1093/ije/dyx102 PMC5837715 29040600

[35] Bowden J Davey Smith G Burgess S. Mendelian randomization with invalid instruments: effect estimation and bias detection through Egger regression. Int J Epidemiol (2015) 44:512–25. doi: 10.1093/ije/dyv080 PMC4469799 26050253

[36] Ni JJ Xu Q Yan SS Han BX Zhang H Wei XT. Gut microbiota and psychiatric disorders: A two-sample mendelian randomization study. Front Microbiol (2021) 12:737197. doi: 10.3389/fmicb.2021.737197 35185808 PMC8856606

[37] Verbanck M Chen CY Neale B Do R. Detection of widespread horizontal pleiotropy in causal relationships inferred from Mendelian randomization between complex traits and diseases. Nat Genet (2018) 50:693–98. doi: 10.1038/s41588-018-0099-7 PMC6083837 29686387

[38] Xiang K Wang P Xu Z Hu YQ He YS Chen Y. Causal effects of gut microbiome on systemic lupus erythematosus: A two-sample mendelian randomization study. Front Immunol (2021) 12:667097. doi: 10.3389/fimmu.2021.667097 34557183 PMC8453215

[39] Pierce BL Ahsan H Vanderweele TJ. Power and instrument strength requirements for Mendelian randomization studies using multiple genetic variants. Int J Epidemiol (2011) 40:740–52. doi: 10.1093/ije/dyq151 PMC3147064 20813862

[40] Mazidi M Shekoohi N Covic A Mikhailidis DP Banach M. Adverse Impact of Desulfovibrio spp. and Beneficial Role of Anaerostipes spp. on Renal Function: Insights from a Mendelian Randomization Analysis. Nutrients (2020) 12:2216. doi: 10.3390/nu12082216 32722370 PMC7468709

[41] Wang X Yang S Li S Zhao L Hao Y Qin J. Aberrant gut microbiota alters host metabolome and impacts renal failure in humans and rodents. Gut (2020) 69:2131–42. doi: 10.1136/gutjnl-2019-319766 PMC7677483 32241904

[42] Mahmoodpoor F Rahbar Saadat Y Barzegari A Ardalan M Zununi Vahed S. The impact of gut microbiota on kidney function and pathogenesis. BioMed Pharmacother (2017) 93:412–19. doi: 10.1016/j.biopha.2017.06.066 28654798

[43] Zhu Y He H Tang Y Peng Y Hu P Sun W. Reno-protective effect of low protein diet supplemented with α-ketoacid through gut microbiota and fecal metabolism in 5/6 nephrectomized mice. Front Nutr (2022) 9:889131. doi: 10.3389/fnut.2022.889131 35845811 PMC9280408

[44] Guirong YE Minjie Z Lixin YU Junsheng YE Lin Y Lisha S. [Gut microbiota in renal transplant recipients, patients with chronic kidney disease and healthy subjects]. Nan Fang Yi Ke Da Xue Xue Bao (2018) 38:1401–08. doi: 10.12122/j.issn.1673-4254.2018.12.01 PMC6744200 30613005

[45] Lai L Li Y Liu J Luo L Tang J Xue J. Bovine serum albumin aggravates macrophage M1 activation and kidney injury in heterozygous Klotho-deficient mice via the gut microbiota-immune axis. Int J Biol Sci (2021) 17:742–55. doi: 10.7150/ijbs.56424 PMC7975693 33767585

[46] Szeto CC McIntyre CW Li PK. Circulating bacterial fragments as cardiovascular risk factors in CKD. J Am Soc Nephrol (2018) 29:1601–08. doi: 10.1681/asn.2018010068 PMC6054355 29666156

[47] Andrade-Oliveira V Amano MT Correa-Costa M Castoldi A Felizardo RJ de Almeida DC. Gut bacteria products prevent AKI induced by ischemia-reperfusion. J Am Soc Nephrol (2015) 26:1877–88. doi: 10.1681/asn.2014030288 PMC4520159 25589612

[48] Hu X Ouyang S Xie Y Gong Z Du J. Characterizing the gut microbiota in patients with chronic kidney disease. Postgrad Med (2020) 132:495–505. doi: 10.1080/00325481.2020.1744335 32241215

[49] Sallée M Dou L Cerini C Poitevin S Brunet P Burtey S. The aryl hydrocarbon receptor-activating effect of uremic toxins from tryptophan metabolism: a new concept to understand cardiovascular complications of chronic kidney disease. Toxins (Basel) (2014) 6:934–49. doi: 10.3390/toxins6030934 PMC3968369 24599232

[50] Jiang S Xie S Lv D Wang P He H Zhang T. Alteration of the gut microbiota in Chinese population with chronic kidney disease. Sci Rep (2017) 7:2870. doi: 10.1038/s41598-017-02989-2 28588309 PMC5460291

[51] Zhao J Ning X Liu B Dong R Bai M Sun S. Specific alterations in gut microbiota in patients with chronic kidney disease: an updated systematic review. Ren Fail (2021) 43:102–12. doi: 10.1080/0886022x.2020.1864404 PMC7808321 33406960

[52] Gibson GR. Physiology and ecology of the sulphate-reducing bacteria. J Appl Bacteriol (1990) 69:769–97. doi: 10.1111/j.1365-2672.1990.tb01575.x 2286579

[53] Quigley EMM. Nutraceuticals as modulators of gut microbiota: Role in therapy. Br J Pharmacol (2020) 177:1351–62. doi: 10.1111/bph.14902 PMC7056471 31659751

[54] Feng YL Cao G Chen DQ Vaziri ND Chen L Zhang J. Microbiome-metabolomics reveals gut microbiota associated with glycine-conjugated metabolites and polyamine metabolism in chronic kidney disease. Cell Mol Life Sci (2019) 76:4961–78. doi: 10.1007/s00018-019-03155-9 PMC11105293 31147751

[55] Mishima E Fukuda S Mukawa C Yuri A Kanemitsu Y Matsumoto Y. Evaluation of the impact of gut microbiota on uremic solute accumulation by a CE-TOFMS-based metabolomics approach. Kidney Int (2017) 92:634–45. doi: 10.1016/j.kint.2017.02.011 28396122

[56] Brinkkoetter PT Ising C Benzing T. The role of the podocyte in albumin filtration. Nat Rev Nephrol (2013) 9:328–36. doi: 10.1038/nrneph.2013.78 23609563

[57] Hoshino J Furuichi K Yamanouchi M Mise K Sekine A Kawada M. A new pathological scoring system by the Japanese classification to predict renal outcome in diabetic nephropathy. PloS One (2018) 13:e0190923. doi: 10.1371/journal.pone.0190923 29408865 PMC5800536

[58] Watkins EB Phillips RS. Inhibition of tyrosine phenol-lyase from Citrobacter freundii by 2-azatyrosine and 3-azatyrosine. Biochemistry (2001) 40:14862–8. doi: 10.1021/bi015707s 11732906

[59] Carrero JJ Stenvinkel P. Inflammation in end-stage renal disease–what have we learned in 10 years? Semin Dial (2010) 23:498–509. doi: 10.1111/j.1525-139X.2010.00784.x 21039875

[60] Lau WL Savoj J Nakata MB Vaziri ND. Altered microbiome in chronic kidney disease: systemic effects of gut-derived uremic toxins. Clin Sci (Lond) (2018) 132:509–22. doi: 10.1042/cs20171107 29523750

[61] Tsai YL Lin TL Chang CJ Wu TR Lai WF Lu CC. Probiotics, prebiotics and amelioration of diseases. J BioMed Sci (2019) 26:3. doi: 10.1186/s12929-018-0493-6 30609922 PMC6320572

[62] Vaziri ND Wong J Pahl M Piceno YM Yuan J DeSantis TZ. Chronic kidney disease alters intestinal microbial flora. Kidney Int (2013) 83:308–15. doi: 10.1038/ki.2012.345 22992469

[63] Hu Y Chen D Zheng P Yu J He J Mao X. The bidirectional interactions between resveratrol and gut microbiota: an insight into oxidative stress and inflammatory bowel disease therapy. BioMed Res Int (2019) 2019:5403761. doi: 10.1155/2019/5403761 31179328 PMC6507241

[64] Li YJ Chen X Kwan TK Loh YW Singer J Liu Y. Dietary Fiber Protects against Diabetic Nephropathy through Short-Chain Fatty Acid-Mediated Activation of G Protein-Coupled Receptors GPR43 and GPR109A. J Am Soc Nephrol (2020) 31:1267–81. doi: 10.1681/asn.2019101029 PMC7269358 32358041

[65] Frosali S Pagliari D Gambassi G Landolfi R Pandolfi F Cianci R. How the intricate interaction among toll-like receptors, microbiota, and intestinal immunity can influence gastrointestinal pathology. J Immunol Res (2015) 2015:489821. doi: 10.1155/2015/489821 26090491 PMC4452102

[66] Bruning EE Coller JK Wardill HR Bowen JM. Site-specific contribution of Toll-like receptor 4 to intestinal homeostasis and inflammatory disease. J Cell Physiol (2021) 236:877–88. doi: 10.1002/jcp.29976 32730645

